# Combination of Dexmedetomidine and Ketamine for Magnetic Resonance Imaging Sedation

**DOI:** 10.3389/fneur.2019.00416

**Published:** 2019-04-24

**Authors:** Joong-Goo Kim, Han-Bin Lee, Sang-Beom Jeon

**Affiliations:** ^1^Department of Neurology, Jeju National University Hospital, Jeju, South Korea; ^2^Department of Neurology, Asan Medical Center, University of Ulsan College of Medicine, Seoul, South Korea

**Keywords:** magnetic resonance imaging, sedation, complications, midazolam, dexmedetomidine, ketamine

## Abstract

**Objectives:** The use of dexmedetomidine and ketamine (DEX–KET) combination for magnetic resonance imaging (MRI) sedation has not been evaluated. We investigated the efficacy and safety of DEX–KET for sedation of patients undergoing MRI of the brain.

**Methods:** This quasi-experimental study was conducted to compare the DEX–KET combination and midazolam for MRI sedation. We included 72 patients undergoing brain MRI following bolus injection of midazolam or DEX–KET. In August 1, 2016 a new MRI sedation protocol was implemented. After protocol implementation, bolus doses of DEX–KET were administered (DEX–KET group). Thirty-six patients from the MIDA group and 36 patients from the DEX–KET group underwent MRI sequences and were compared regarding the MRI scan time and sedation-related complications (desaturation, hypotension, cardiorespiratory arrest, and aspiration pneumonia).

**Results:** All MRI sequences were completed for 30 patients (83.3%) from the MIDA group and for 33 patients (91.7%) from the DEX–KET group (*P* = 0.476). The median MRI scan time was 100.0 min (interquartile range, 87.0–111.5 min) in the MIDA group and 53.5 min (interquartile range, 45.0–60.5 min) in the DEX–KET group (*P* < 0.001). Complications occurred in 24 (66.7%) and 8 (22.2%) patients of the MIDA and DEX–KET group, respectively (*P* < 0.001).

**Conclusions:** The efficacy of DEX–KET sedation was comparable to that of midazolam for MRI examination. DEX–KET was related to shorter scan time and lower occurrence of complications compared to midazolam.

## Introduction

Neurocritically ill patients often require brain magnetic resonance imaging (MRI) in addition to conventional neurological evaluations. Brain MRI can reveal structural lesions with a high sensitivity due to its excellent spatial resolution and enhanced soft tissue contrast (1–3). To acquire MRI images, patients' cooperation is imperative. However, keeping patients with brain dysfunction immobilized in a dark, noisy MRI scanner is challenging and may require administration of sedative agents to ensure motion-artifact-free images (4–8). The use of sedative agents may not always guarantee patients' cooperation and it may even exert side effects. In addition, prolonged MRI duration due to uncooperative patients may increase the occurrence of adverse events due to withheld care management upon absence from the neurological intensive care unit (neuro-ICU) (9). Therefore, a proper sedation regimen is crucial to manage neurocritically ill patients undergoing MRI.

Dexmedetomidine, an α-2 adrenoreceptor agonist, has been widely used in intensive care units due to its beneficial effects in conscious sedation, anxiety relief, and pain control, associated with minimal or absent respiratory depression (10). However, the use of dexmedetomidine for sedation of neurocritically ill patients undergoing MRI has several limitations, such as a delayed onset of sedative action, and cardio-inhibitory effects such as hypotension and bradycardia (10). Ketamine, a phencyclidine analog and antagonist of the N-methyl-d-aspartate receptor, is characterized by a rapid-onset, short-acting sedation effect and preservation of airway reflexes (11). Opposite to dexmedetomidine, ketamine exerts cardio-stimulatory effects including hypertension and tachycardia (11). Thus, a combination of dexmedetomidine and ketamine (DEX–KET) might counterbalance the side effects and enhance the beneficial effects of each drug. However, to our knowledge, the use of DEX–KET has not been evaluated for MRI sedation.

This study investigated the efficacy and safety of DEX–KET for MRI sedation in neurocritically ill patients.

## Methods

### Study Population

This is quasi-experimental study to evaluate efficacy and safety of DEX–KET for MRI sedation in neurocritically ill patients compared with midazolam for MRI sedation. It was performed at the Asan Medical Center, Seoul, Korea, between August 1, 2014 and October 31, 2017. According to our management protocol, brain MRI was considered for all patients with presumed brain diseases, excluding those who did not have diagnostic equipoise or did not need follow-up scans following previous computed tomography or MRI before admission to the neuro-ICU. MRI was also not performed when patients or their proxy did not consent or when a medical condition contraindicated an MRI scan. In this study, we included consecutive patients who (1) were ≥18 years of age; (2) underwent brain MRI at admission to the neuro-ICU; and (3) received midazolam or DEX–KET for MRI sedation. We excluded patients when (1) they were receiving continuous infusion of sedative agents for critical care in the neuro-ICU; (2) they underwent follow-up MRIs; and (3) they underwent other diagnostic studies outside the neuro-ICU at the time of transportation to the MRI room.

This study was approved by the institutional review board of the Asan Medical Center, and the need for written informed consent was waived due to the retrospective nature of the study.

### Sedation Protocol

On August 1, 2016, we implemented a written protocol for MRI sedation for the first time. Until then, MRI sedation was performed by routinely preparing a bolus dose (2–3 mg) of midazolam, when patients were transported to the MRI room and administering it intravenously if the patients behaved (or were expected to behave) uncooperatively. If the sedation effects of the injection proved insufficient, additional doses of midazolam were administered up to 10 mg, provided that the patients' vitals were stable. For this study, the patients who received this type of sedation were defined as the MIDA group. Treating physician and/or interns routinely accompanied patients while the patients were transported between the neuro-ICU and the MRI room. During performing MRI, the patient's vital signs including heart rate, respiratory rate, and oxygen saturation were routinely monitored. Following implementation of the new MRI sedation protocol, patients who need MRI are routinely screened for the following criteria, which indicate the need for sedation: (1) agitation [the Richmond Agitation-Sedation Scale (RASS) ≥1] over the preceding 24 h, (2) need for physical restraints, (3) recent failure to complete a neuroimaging study, and (4) recent need for sedatives in neuroimaging studies. When patients met the criteria for MRI sedation, they were further screened for the following criteria, exclusive of DEX–KET administration: (1) QTc prolongation > 550 ms on electrocardiography, (2) second- or third-degree atrioventricular block on electrocardiography, (3) severe heart failure, (4) intractable hypertension or hypotension, and (5) allergy to dexmedetomidine or ketamine. Patients meeting the inclusion criteria for MRI and not the exclusion criteria for DEX–KET were concurrently administered 35 μg of dexmedetomidine diluted in 0.9% saline intravenously over 10 min and 35 mg of ketamine diluted in 0.9% saline intravenously over 1 min using two different intravenous lines. Such bolus administration of DEX–KET was immediately performed before the patients left the neuro-ICU (Supplementary Table 1, Supplementary Figure 1). Additional doses of DEX–KET and other sedative agents could be administered in the MRI room at the discretion of the attending physician if the sedation effects were not apparent. For the present study, patients who received a combination of dexmedetomidine and ketamine following implementation of the new MRI sedation protocol were defined as the DEX–KET group. DEX–KET was administered according to the protocol's inclusion criteria, whereas midazolam was administered at the discretion of the attending physician. For further analysis, a third group was defined, including the patients who were not assessed for non-cooperation *a priori* but met the indications for preemptive sedation when assessed *a posteriori*. This group was named MIDA', and the results for the MIDA' group were compared to those of the DEX–KET group.

### Clinical Assessment and Data Acquisition

Following implementation of the new protocol, the following data were prospectively collected from our registry: inclusion and exclusion criteria for MRI sedation, RASS core, systolic and diastolic blood pressure, heart rate, respiratory rate, and oxygen saturation on pulse oximeter. Parameters subject to routine hourly measure included vital signs, RASS score, and Glasgow Coma Scale score. Other data extracted from the electronic medical records included demographic information, comorbidities including hypertension, diabetes mellitus, hypercholesterolemia, heart disease, previous stroke, history of smoking or alcohol consumption, scores from the Acute Physiology and Chronic Health Enquiry II (APACHE II), diagnosis at admission to the neuro-ICU, and the length of neuro-ICU stay.

MRI sedation was considered successful when the patient completed all ordered MRI sequences. We defined the MRI scan time as the time difference between the times of departure from and return to the neuro-ICU. Detailed information concerning the orders and acquisitions of MRI sequences was retrieved from the electronic medical records and the picture archive and communication system of the hospital. Vital signs were serially evaluated for the following time points: time 0, immediately before administration of sedative agents (midazolam or DEX–KET); time 1, immediately after bolus infusion of sedative agents; time 2, time of return to the neuro-ICU following MRI. Although we routinely checked vital signs multiple times and continuously monitored oxygen saturation using pulse oximeter while patients were out of the neuro-ICU, we documented vital signs only at aforementioned three time points. Thus, data on vital signs for the current study were obtained at these three time points which were documented on electronic medical records and the protocol sheet (Supplementary Figure 1).

### Complications

Sedation-related complications included oxygen desaturation, hypotension, cardiorespiratory arrest, and aspiration pneumonia. Oxygen desaturation was defined as a decrease in oxygen saturation to <90%. Hypotension was defined as a 40 mmHg decrease in systolic and/or diastolic blood pressure or the need to use inotropic agents within an hour following MRI sedation. Cardiorespiratory arrest was defined as a patient's need for endotracheal intubation or chest compressions within an hour following MRI sedation. Aspiration pneumonia was defined as the presence of a new or progressive radiographic infiltrate plus at least two of the following three clinical features: body temperature > 38.0°C, leukocytosis or leukopenia, and purulent secretions, developing within 1 week following MRI sedation.

### Statistical Analyses

The DEX–KET group was compared with the MIDA and MIDA' groups in terms of the baseline characteristics, clinical status, and vital signs before and after MRI sedation, as well as the MRI scan time, success in obtaining ordered MRI sequences, and occurrence of complications. In addition, patients with and without sedation-related complications were compared in terms of baseline characteristics, clinical status, and sedation regimen.

Univariate analyses were performed using the Pearson chi-square test, Fisher's exact test, Student's *t*-test, Mann–Whitney *U*-test, or Jonckheere-Terpstra test, as appropriate. Multivariate logistic regression analysis was performed to identify the independent contribution of each variable for the development of sedation-related complications. Variables with a *P* < 0.2 in the univariate analysis were included as candidate variables in the multivariate analysis and removed by backward stepwise selection. Additional analysis using forward selection confirmed the final model. A two-tailed *P* < 0.05 was considered indicative of significant differences in all statistical analyses. All statistical analyses were performed with SPSS version 21.0 (IBM, Armonk, NY, USA).

## Results

**Figure 1:**
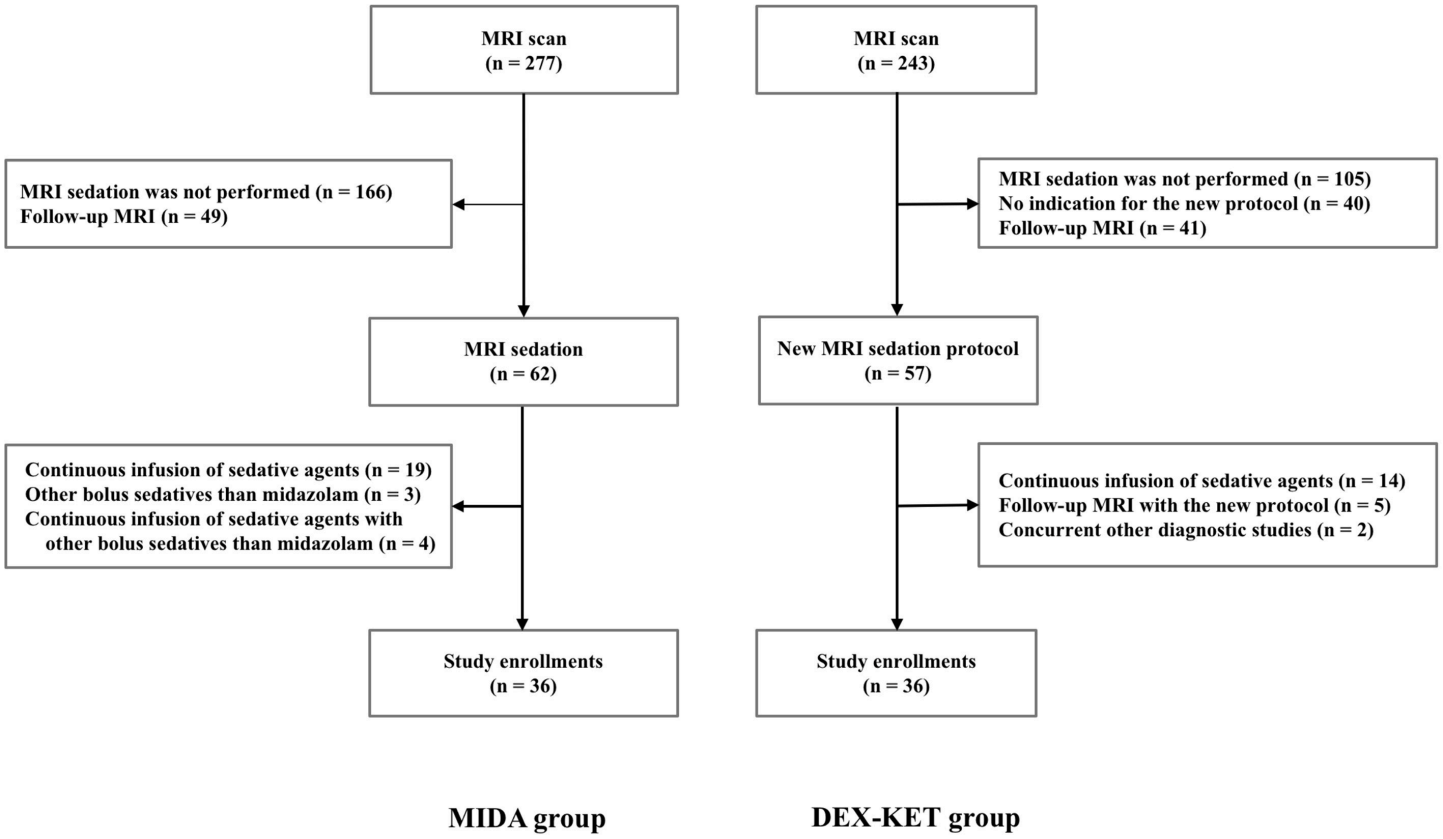
Flowchart of the patient selection process.

A total of 1,342 patients were admitted to our neuro-ICU between August 1, 2014 and October 31, 2017. Prior to protocol implementation, 277 patients underwent MRI examination. Of these, 36 patients met the criteria for inclusion in the MIDA. The remaining patients were excluded based on the following reasons: no need for MRI sedation (*n* = 166); need for follow-up MRI scan (*n* = 49); and need for continuous infusion of sedative agents for critical care and/or other agents than midazolam (*n* = 26). Among the 36 patients in the MIDA group, 26 were further included in the MIDA' group (Supplementary Figure 2). Following implementation of the new protocol, 243 patients underwent MRI examination, and 36 met the criteria to integrate the DEX–KET. The remaining patients were excluded due to the following reasons: no need for MRI sedation (*n* = 105); MRI sedation without indication for the new protocol (*n* = 40); follow-up MRI (*n* = 46); need for continuous infusion of sedative agents for critical care (*n* = 14); and need for other concurrent diagnostic studies (*n* = 2) (Figure 1). The patient's median age was 69 years [interquartile range (IQR), 54.5–78.0 years], and 36 (50%) were men. The median body weight was 64.0 kg (IQR, 53.5–71.7 kg), and the median time interval between admission to the neuro-ICU and MRI examination was 2.0 days (IQR, 1.0–4.0 days) (Table 1).

**Table 1:** Baseline characteristics.

	**MIDA group (*n* = 36)**	**DEX–KET group (*n* = 36)**	***P-*value**
**Demographics**			
Age, years	67.0 (51.5–78.5)	72.0 (56.0–77.0)	0.454
Sex, male	22 (61.1)	14 (38.9)	0.099
Body mass index, kg/m^2^	24.4 (22.7–26.7)	24.1 (21.9–26.2)	0.551
**Comorbidities and risk factors**			
Hypertension	18 (50.0)	21 (58.3)	0.636
Diabetes mellitus	11 (30.6)	11 (30.6)	1.000
Hypercholesterolemia	6 (16.7)	4 (11.1)	0.733
Atrial fibrillation	9 (25.0)	12 (33.3)	0.604
Coronary artery disease	5 (13.9)	3 (8.3)	0.708
Previous stroke	6 (16.7)	5 (13.9)	1.000
Cancer	3 (8.3)	3 (8.3)	1.000
Smoking	11 (30.6)	13 (36.1)	0.803
Alcohol consumption	13 (36.1)	10 (27.8)	0.613
**Diagnosis on admission**			
Stroke	22 (61.1)	30 (83.3)	0.066
CNS infection	7 (19.4)	4 (11.1)	0.512
Seizure or status epilepticus	5 (13.9)	2 (5.6)	0.426
Demyelinating disease	2 (5.6)	0 (0)	0.473
**Clinical status on admission**			
Glasgow Coma Scale	12.0 (10.0–14.0)	12.0 (10.0–14.0)	0.624
APACHE-II score	13.0 (10.0–7.0)	15.5 (12.5–19.5)	0.102

*MIDA, midazolam; DEX–KET, combination of dexmedetomidine and ketamine; CNS, central nervous system; APACHE-II, Acute Physiology and Chronic Health Enquiry II; MRI, magnetic resonance imaging*.

*Results presented as numbers (%) or medians (interquartile range)*.

### MRI Sedation

All patients within the MIDA group received 2–3 mg of midazolam upon arrival to the MRI room, whereas all patients within the DEX–KET group received 35 μg of dexmedetomidine and 35 mg of ketamine in the neuro-ICU, immediately before transportation to the MRI room. In both groups, additional sedative agents were administered in the MRI room according to the judgment of the treating physician. Eleven patients in the MIDA group received additional dosages of midazolam up to a maximum of 10 mg. Within the DEX–KET group, 10 patients needed additional medication, 7 of which received an additional combination of 35 μg of dexmedetomidine and 35 mg of ketamine, 2 received midazolam, and 1 received lorazepam. Data on medication doses in MIDA and DEX-KET groups are shown in Supplementary Table 2, and data on medication doses in MIDA' and DEX-KET groups are shown in Supplementary Table 3. Reasons for requiring MRI sedation are shown in Supplementary Tables 4, 5. RASS changes following sedative administration are shown in Supplementary Figure 3.

### Hemodynamic Changes

At time 0, no significant differences were found between the MIDA and DEX–KET groups regarding the vital signs. At time 1, the systolic blood pressure was marginally higher in the DEX–KET group than that in the MIDA group, and at time 2, the respiratory rate was significantly lower in the DEX–KET than that in the MIDA group, whereas the heart rate was marginally higher in MIDA than that in the DEX–KET group. The heart rate (*P* = 0.037), respiratory rate (*P* = 0.022), and systolic blood pressure (*P* = 0.058) showed decreasing trends following administration of DEX–KET but not of midazolam (Table 2).

**Table 2:** Sedation-related changes in vital signs.

	**MIDA group (*n* = 36)**	***P*-value^a^**	**DEX–KET group (*n* = 36)**	***P-*value^b^**	***P*-value^c^**
Systolic blood pressure		0.252		0.058	
Time 0	131.0 (117–146.0)		133.5 (124.5–148.0)		0.195
Time 1	131.5 (114.5–145.5)		135.5 (127.5–157.5)		0.064
Time 2	122.0 (109.0–140.5)		128.5 (113.5–143.0)		0.427
Diastolic blood pressure		0.625		0.267	
Time 0	72.5 (63.0–80.5)		75.0 (61.5–86.0)		0.562
Time 1	76.0(65.5–82.0)		76.0 (62.0–87.5)		0.535
Time 2	69.0 (62.5–78.5)		70.0 (58.5–80.0)		0.991
Heart rate		0.426		0.037	
Time 0	82.5 (71.0–95.5)		85.5 (66.0–94.5)		0.744
Time 1	87.0 (71.0–97.5)		79.5 (66.0–92.5)		0.186
Time 2	82.0 (67.5–92.5)		71.0 (59.0–87.5)		0.064
Respiratory rate		0.221		0.022	
Time 0	18.0 (14.5–21.5)		19.0 (17.0–22.0)		0.180
Time 1	20.0 (18.0–21.0)		19.0 (16.5–23.0)		0.888
Time 2	19.5 (18.0–21.5)		16.5 (14.0–20.5)		0.011
Oxygen saturation		0.348		0.904	
Time 0	98.0 (96.0–100.0)		98.0 (96.0–99.5)		0.863
Time 1	97.0 (95.0–98.0)		97.0 (96.0–99.5)		0.301
Time 2	99.0 (97.5–99.5)		98.0 (96.0–99.5)		0.380

*MIDA, midazolam; DEX–KET, combination of dexmedetomidine and ketamine*.

*Time 0 = immediately before administration of sedative agents; time 1 = immediately after bolus infusion of sedative agents; time 2 = soon after returning to the neurological intensive care unit*.

*Results presented as a median (interquartile range)*.

a *P for trends from time 0 to time 2 in MIDA group for each vital sign*.

b *P for trends from time 0 to time 2 in DEX–KET group for each vial sign*.

c *P-value for difference of each vital sign between MIDA group and DEX–KET group for each time point*.

### Outcome and Complications

While the two groups presented a similar MRI sedation success rate (83.3% in MIDA group vs. 91.7% in DEX–KET group; *P* = 0.476), the median MRI scan time was significantly shorter in DEX–KET group as compared with MIDA group [100.0 min (IQR, 87.0–111.5 min) in MIDA group vs. 53.5 min (IQR, 45.0–60.5 min) in DEX–KET group; *P* < 0.001]. No differences were observed between the groups concerning the length of neuro-ICU stay.

The DEX–KET group presented a lower complication rate than the MIDA group (66.7% in MIDA group vs. 22.2% in DEX–KET group; *P* < 0.001), including oxygen desaturation (16.7% in MIDA group vs. 2.8% in DEX–KET group), hypotension (5.6% in MIDA group vs. 0% in DEX–KET group), cardiorespiratory arrest (0% in both groups), and aspiration pneumonia (61.1% in MIDA group vs. 19.4% in DEX–KET group) (Table 3). History of smoking (*P* = 0.022) and initial diagnosis of stroke (*P* = 0.009) was associated with a higher risk of complications following MRI sedation. MRI sedation with DEX–KET as compared with midazolam was associated with lower risk for sedation-related complications (OR, 0.14; 95% CI, 0.05–0.41; *P* < 0.001), and this result was confirmed by multivariate analysis (OR, 0.13; 95% CI, 0.04–0.41; *P* = 0.001) (Table 4). Further analyses comparing the MIDA' and DEX–KET groups showed similar results (Supplementary Tables 6–11).

**Table 3:** Outcomes and complications according to the MRI sedation protocol.

	**MIDA group (*n* = 36)**	**DEX–KET group (*n* = 36)**	***P*-value**
Outcomes relevant to MRI sedation			
Success in MRI sedation	30 (83.3)	33 (91.7)	0.476
MRI scan time, min	100.0 (87.0–111.5)	53.5 (45.0–60.5)	<0.001
Length of neuro-ICU stay, day	4.0 (2.0–6.0)	4.0 (3.0–6.0)	0.373
Complications following MRI sedation	24 (66.7)	8 (22.2)	<0.001
Oxygen desaturation	6 (16.7)	1 (2.8)	0.112
Hypotension	2 (5.6)	0 (0)	0.473
Cardiorespiratory arrest	0 (0)	0 (0)	1.000
Aspiration pneumonia	22 (61.1)	7 (19.4)	0.001

*MRI, magnetic resonance imaging; neuro-ICU, neurological intensive care unit*.

*Results presented as numbers (%) or medians (interquartile ranges)*.

**Table 4:** Factors associated with sedation-related complications.

	**Univariable analysis**	***P*-value**	**Multivariable analysis**	***P-*value**
	**Odds ratio (95% CI)**		**Adjusted odds ratio (95% CI)**	
**Demographics**				
Age, years	1.000 (0.97–1.03)	0.852		
Sex, male	1.25 (0.49–3.18)	0.635		
Body mass index, kg/m^2^	0.98 (0.86–1.12)	0.783		
**Comorbidities and risk factors**				
Hypertension	0.93 (0.37–2.36)	0.874		
Diabetes mellitus	0.62 (0.22–1.74)	0.360		
Hypercholesterolemia	2.08 (0.53–8.11)	0.293		
Atrial fibrillation	0.52 (0.18–1.50)	0.227		
Coronary artery disease	0.72 (0.16–3.30)	0.676		
Previous stroke	0.41 (0.10–1.71)	0.223		
Cancer	2.71 (0.46–15.87)	0.268		
Smoking	0.28 (0.10–0.83)	0.022	0.34 (0.09–1.25)	0.105
Alcohol consumption	0.56 (0.20–1.55)	0.261		
**Diagnosis on admission**				
Stroke	0.23 (0.07–0.69)	0.009		
CNS infection	4.11 (0.99–17.06)	0.051	4.54 (0.83–24.83)	0.081
Seizure or status epilepticus	1.90 (0.39–9.20)	0.481		
Demyelinating disease				
**Clinical status on admission**				
Glasgow Coma Scale	0.83 (0.68–1.01)	0.061	0.81 (0.63–1.04)	0.960
APACHE-II score	1.04 (0.95–1.13)	0.400		
MRI sedation, DEX–KET	0.14 (0.05–0.41)	<0.001	0.13 (0.04–0.41)	0.001

*DEX–KET, combination of dexmedetomidine and ketamine; CNS, central nervous system; APACHE-II, Acute Physiology and Chronic Health Enquiry II; MRI, magnetic resonance imaging*.

## Discussion

This study investigated the efficacy and safety of a newly implemented sedation protocol using a combination of dexmedetomidine and ketamine administered intravenously to perform brain MRI in neurocritically ill patients. A bolus dose of DEX–KET was infused over 10 min immediately before the patients were taken from the ICU into the MRI room. This preemptive sedation was conceived for uncooperative patients at a high risk of failure to complete ordered MRI sequences. Successful MRI examination was achieved in 92% of patients, although 28% of the patients required additional sedative doses. With the new sedation protocol, the median MRI scan time decreased from 100 to 54 min. Moreover, DEX–KET was related to a significantly lower rate of sedation-related complications, including oxygen desaturation, hypotension, and aspiration pneumonia, as compared with a use of midazolam. Thus, the implementation of a protocol which preemptively administer a combination of dexmedetomidine and ketamine in patients with high risks of MRI failure can be considered effective and safe.

Lack of patients' cooperation is the most common reason for failure to complete MRI examinations (12, 13). It often occurs unexpectedly in the MRI room, even among patients who are usually cooperative or those who were sedated while in the ICU (7). This phenomenon is particularly prominent in patients with brain disorders. Failure to complete all ordered MRI sequences may cause the physician to miss the diagnosis, leading to failure to provide appropriate treatment. On the contrary, when a patient spends a long time in the MRI room to complete all ordered MRI sequences, the patient may be exposed to an increased risk of complications because critical care is withheld during the time spent away from the ICU. Sedative agents *per se* may also result in serious side effects. Thus, a preemptive, effective, and safe MRI sedation protocol is necessary for a successful management of critically ill patients.

Our MRI sedation protocol comprises preemptive administration of DEX–KET in the ICU prior to patients' transportation into the MRI room. In the present study, all patients presented stable vital signs prior to transportation into the MRI room. One patient in the DEX–KET group developed oxygen desaturation in the MRI room, whereas six patients in the MIDA group experienced the same complication. Episodes of hypotension occurred in two patients of the MIDA group, but none in the DEX–KET group. Aspiration pneumonia occurred in 22 patients of the MIDA group, but only 7 in the DEX–KET group. Taken together, these results suggest that DEX–KET is safer than midazolam. However, 28% of the patients receiving DEX–KET required additional sedation, which may have been related to the low initial dose, which may be insufficient for some patients. In fact, our protocol uses a fixed dose of DEX–KET (35 μg of dexmedetomidine plus 35 mg of ketamine), whereas the usual bolus doses of dexmedetomidine and ketamine are 1 μg/kg for ICU sedation and 1–2 mg/kg for procedural sedation, respectively (14–16). The median of the patients' body weight was 64 kg; therefore, the bolus dose of DEX–KET used in the current study was much lower than usual. The fact that a fixed dose regimen was adopted to avoid medication errors as well as to reduce the workload involved in the preparation of DEX–KET individual doses. Because the dose of each drug was low, a drug combination was used. Although the need for additional sedation could be due to DEX–KET having a shorter duration than expected, further studies are needed to investigate the optimal doses of DEX–KET for MRI sedation.

In addition to the inherent limitations of a small retrospective design, the present study has some limitations. First, midazolam was administered at the discretion of treating physicians, but DEX–KET was administered according to predefined indications. Thus, the better outcomes of the DEX–KET group as opposed to the MIDA group could result from not only the choice of drugs but also the different sedation instructions followed for each group. However, the subgroup analysis comparing the MIDA' (patients who were not assessed for non-cooperation *a priori* but met the indications for preemptive sedation when assessed *a posteriori*) and DEX–KET groups provided similar results. Second, midazolam was administered in the MRI room, but DEX–KET was administered in the ICU. The saving of time for infusion of sedative agents before transportation to the MRI room, however, would not affect main results of the current study, because further analysis still showed a statistical significance for shorter MRI scan time after addition of 10 min (infusion time) in the DEX-KET group [100.0 min (IQR, 87.0–111.5 min) vs. 63.5 min (IQR, 55.0–70.5 min); *P* < 0.001]. Actually, adoption of the new MRI sedation protocol was a bundle approach to screen uncooperative patients at risk to develop complications as well as to apply a presumably better drug regimen. Previous studies have demonstrated that protocolized critical care improves patient outcome (17). We suggest that routine screening for patients requiring MRI sedation is necessary because a substantial number of patients behave uncooperatively, leading to delays, failure, or complications during MRI examination. Further studies are needed to investigate uncooperative patients during MRI examination. Third, occurrence of delirium after MRI sedation—a major side effect of sedative agents—was not assessed. However, previous studies have shown that patients exposed to dexmedetomidine experience less delirium than those exposed to midazolam (18).

In conclusion, a protocolized sedation with DEX–KET was comparable to a non-protocolized sedation with midazolam for completion of ordered MRI sequences. However, the DEX–KET protocol was related to a shorter scan time and less complications than midazolam. A protocolized administration of DEX–KET for MRI sedation of potentially uncooperative patients is potentially effective and safe.

## Ethics Statement

Ethical approval was granted according to national requirements, and the need for written informed consent was waived (Institutional Review Board at Asan Medical Center: 2016-1262).

## Author Contributions

JGK, HBL, and SBJ contributed to the article by participating in the concept and design, acquisition of data, and critical revision of the manuscript for intellectual content. JGK and SBJ analysis and interpretation of data and drafting of the manuscript.

### Conflict of Interest Statement

The authors declare that the research was conducted in the absence of any commercial or financial relationships that could be construed as a potential conflict of interest.

## References

[1] Theophilus SC Kandasamy R Bakar KA Abdullah JM Neuroimaging in the neuro-ICU. In: Wartenberg KE Shukri K Abdelhak T, editors. Neurointensive Care. 1st ed. Switzerland: Springer International Publishing (2015). p. 299–312.

[2] Kastrup O Wanke I Maschke M. Neuroimaging of infections. NeuroRx. (2005) 2:324–32. 10.1602/neurorx.2.2.324 15897953 PMC1064994

[3] Kuzniecky RI. Neuroimaging of epilepsy: therapeutic implications. NeuroRx. (2005) 2:384–93. 10.1602/neurorx.2.2.384 15897958 PMC1064999

[4] Bluemke DA Breiter SN. Sedation procedures in MR imaging: safety, effectiveness, and nursing effect on examinations. Radiology. (2000) 216:645–52. 10.1148/radiology.216.3.r00se45645 10966690

[5] Eshed I Althoff CE Hamm B Hermann KG. Claustrophobia and premature termination of magnetic resonance imaging examinations. J Magn Reson Imaging. (2007) 26:401–4. 10.1002/jmri.21012 17610281

[6] Saunders DE Thompson C Gunny R Jones R Cox T Chong WK. Magnetic resonance imaging protocols for paediatric neuroradiology. Pediatr Radiol. (2007) 37:789–97. 10.1007/s00247-007-0462-9 17487479 PMC1950216

[7] Oberstein A Meves M Bockenheimer S Schlaps D. Obstacles to the routine use of magnetic resonance imaging--results of a multicenter study for evaluating nuclear magnetic resonance tomography. Digitale Bilddiagn. (1990) 10:10–16. 2191824

[8] Brennan SC Redd WH Jacobsen PB Schorr O Heelan RT Sze GK. Anxiety and panic during magnetic resonance scans. Lancet. (1988) 2:512. 10.1016/S0140-6736(88)90159-6 2900435

[9] Kim YS Lee HJ Jeon SB: Management of pain and agitation for patients in the intensive care unit J Neurocrit Care. (2015) 8:53–65. 10.18700/jnc.2015.8.2.53

[10] Giovannitti JA Thoms SM Crawford JJ. Alpha-2 adrenergic receptor agonists: a review of current clinical applications. Anesth Prog. (2015) 62:31–9. 10.2344/0003-3006-62.1.31 25849473 PMC4389556

[11] Kurdi MS Theerth KA Deva RS. Ketamine: current applications in anesthesia, pain, and critical care. Anesth Essays Res. (2014) 8:283–90. 10.4103/0259-1162.143110 25886322 PMC4258981

[12] Murphy KJ Brunberg JA. Adult claustrophobia, anxiety and sedation in MRI. Magn Reson Imaging. (1997) 15:51–4. 10.1016/S0730-725X(96)00351-7 9084025

[13] Kim JG Ko MA Lee HB Jeon SB. Magnetic resonance imaging in niurocritically ill patients: who fails and how? J Patient Saf. (2018). 10.1097/PTS.0000000000000483. [Epub ahead of print]. 29629931

[14] Yu SB. Dexmedetomidine sedation in ICU. Korean J Anesthesiol. (2012) 62:405–11. 10.4097/kjae.2012.62.5.405 22679535 PMC3366305

[15] Kannikeswaran N Lieh-Lai M Malian M Wang B Farooqi A Roback MG. Optimal dosing of intravenous ketamine for procedural sedation in children in the ED - a randomized controlled trial. Am J Emerg Med. (2016) 34:1347–53. 10.1016/j.ajem.2016.03.064 27216835

[16] Jeon SB Koh Y Choi HA Lee K. Critical care for patients with massive ischemic stroke. J Stroke. (2014) 16:146–60. 10.5853/jos.2014.16.3.146 25328873 PMC4200590

[17] Strøm T Martinussen T Toft P. A protocol of no sedation for critically ill patients receiving mechanical ventilation: a randomised trial. Lancet. (2010) 375:475–80. 10.1016/S0140-6736(09)62072-9 20116842

[18] Riker RR Shehabi Y Bokesch PM Ceraso D Wisemandle W Koura F. Dexmedetomidine vs. midazolam for sedation of critically ill patients: a randomized trial. JAMA. (2009) 301:489–99. 10.1001/jama.2009.56 19188334

