# Supplementary material for: Combination of Dexmedetomidine and Ketamine for Magnetic Resonance Imaging Sedation
Source: Front Neurol. 2019 Apr 24;10:416. doi: 10.3389/fneur.2019.00416 (PMC6492498; doi:10.3389/fneur.2019.00416)
Supplement: Supplementary file 1 [file Data_Sheet_1.pdf]

[Supplementary Data]

Combination of Dexmedetomidine and Ketamine for Magnetic Resonance Imaging Sedation in Neurocritically Ill Patients

**Supplementary table 1**	The Richmond Agitation-Sedation Scale
**Supplementary table 2**	Doses of agents administered in the MIDA and DEX–KET groups
**Supplementary table 3**	Doses of agents administered in the MIDA' and DEX–KET groups
**Supplementary table 4**	Reasons to apply the new MRI sedation protocol in the MIDA and DEX–KET groups
**Supplementary table 5**	Reasons to apply the new MRI sedation protocol in the MIDA' and DEX–KET groups
**Supplementary table 6**	Baseline characteristics of the MIDA' and DEX–KET groups
**Supplementary table 7**	Changes of vital signs in the MIDA' and DEX–KET groups
**Supplementary table 8**	Outcomes and complications according to the MRI sedation protocol in the MIDA' and DEX–KET groups
**Supplementary table 9**	Factors associated with complications following MRI sedation in the MIDA' and DEX–KET groups
**Supplementary table 10**	MRI sequence and number of images in the MIDA and DEX–KET groups
**Supplementary table 11**	MRI sequence and number of images in the MIDA' and DEX–KET groups
**Supplementary figure 1**	New MRI sedation protocol for patients in the neuro-ICU
**Supplementary figure 2**	Flowchart of the patient selection process for the MIDA' and DEX–KET groups
**Supplementary figure 3**	Changes in the level of consciousness before and after administration of sedative agents

[Supplementary table 1] The Richmond Agitation-Sedation Scale

Score	Term	Description
4	Combative	Overtly combative or violent; immediate danger to staff
3	Very agitated	Pulls on or removes tube(s) or catheter(s) or has aggressive behavior toward staff
2	Agitated	Frequent nonpurposeful movement or patient–ventilator dyssynchrony
1	Restless	Anxious or apprehensive but movements not aggressive or vigorous
0	Alert and calm	Spontaneously pays attention to caregiver
−1	Drowsy	Not fully alert, but has sustained (more than 10 seconds) awakening, with eye contact, to voice
−2	Light sedation	Briefly (less than 10 seconds) awakens with eye contact to voice
−3	Moderate sedation	Any movement (but no eye contact) to voice
−4	Deep sedation	No response to voice, but any movement to physical stimulation
−5	Unarousable	No response to voice or physical stimulation

[Reference] Sessler CN, Gosnell MS, Grap MJ, Brophy GM, O'Neal PV, Keane KA, et al.: The Richmond Agitation-Sedation Scale: validity and reliability in adult intensive care unit patients. *Am J Respir Crit Care Med.* 2002, 166(10):1338-1344.

[Supplementary table 2] Doses of agents administered in the MIDA and DEX–KET groups

	MIDA group (n=36)	DEX–KET group (n=36)
Initial dose	Midazolam 2 mg (n = 7)	DEX 35 µg + KET 35 mg (n = 36)
	Midazolam 3 mg (n = 29)	
Additional dose	Midazolam 2 mg (n = 6)	DEX 35 µg + KET 35 mg (n = 5)
	Midazolam 3 mg (n = 3)	DEX 35 µg + KET 35 mg + midazolam 3 mg (n = 1)
	Midazolam 5 mg (n = 1)	DEX 35 µg + KET 35 mg + midazolam 5 mg (n = 1)
	Midazolam 10 mg (n = 1)	Midazolam 3 mg (n = 1)
		Midazolam 5 mg (n = 1)
		Lorazepam 10 mg (n = 1)
Total dose		
Mean	Midazolam 3.8 mg	DEX 41.8 µg + KET 41.8 mg
Median	Midazolam 3 mg	DEX 35 µg + KET 35 mg
Mode	Midazolam 3 mg	DEX 35 µg + KET 35 mg

MRI = magnetic resonance imaging; DEX = dexmedetomidine; KET = ketamine

[Supplementary table 3] Doses of agents administered in the MIDA' and DEX–KET groups

	MIDA' group (n=26)	DEX–KET group (n=36)
Initial dose	Midazolam 2 mg (n = 4)	DEX 35 μg + KET 35 mg (n = 36)
	Midazolam 3 mg (n = 22)	
Additional dose	Midazolam 2 mg (n = 6)	DEX 35 μg + KET 35 mg (n = 5)
	Midazolam 3 mg (n = 2)	DEX 35 μg + KET 35 mg + midazolam 3 mg (n = 1)
	Midazolam 10 mg (n = 1)	DEX 35 μg + KET 35 mg + midazolam 5 mg (n = 1)
		Midazolam 3 mg (n = 1)
		Midazolam 5 mg (n = 1)
		Lorazepam 10 mg (n = 1)
Total dose		
Mean	Midazolam 3.9 mg	DEX 41.8 μg + KET 41.8 mg
Median	Midazolam 3 mg	DEX 35 μg + KET 35 mg
Mode	Midazolam 3 mg	DEX 35 μg + KET 35 mg

MRI = magnetic resonance imaging; DEX = dexmedetomidine; KET = ketamine

[Supplementary table 4] Reasons to apply the new MRI sedation protocol in the MIDA and DEX–KET groups

	MIDA group (n = 36)	DEX–KET group (n = 36)	*P* value
Agitation (RASS scale ≥1) for recent 24 hours	9 (25.0)	29 (80.6)	<0.001
Physical restraints for safety issues	23 (63.9)	24 (66.7)	0.600
Recent failure of neuroimaging study	2 (5.6)	3 (8.3)	1.000
Recent use of sedative agents for neuroimaging studies	0 (0)	3 (8.3)	0.238

RASS = the Richmond Agitation-Sedation Scale

[a]Results presented as numbers (%).

[Supplementary table 5] Reasons to apply the new MRI sedation protocol in the MIDA' and DEX–KET groups

	MIDA' group (n = 26)	DEX–KET group (n = 36)	*P* value
Agitation (RASS scale ≥1) for recent 24 hours	9 (34.6)	29 (80.6)	0.001
Physical restraints for safety issues	21 (80.8)	24 (66.7)	0.159
Recent failure of neuroimaging study	2 (7.7)	3 (8.3)	1.000
Recent use of sedative agents for neuroimaging studies	0 (0)	3 (8.3)	0.363

RASS = the Richmond Agitation-Sedation Scale

[a]Results presented as numbers (%).

[Supplementary table 6] Baseline characteristics of the MIDA' and DEX–KET groups

	MIDA' group (n = 26)	DEX–KET group (n = 36)	*P* value
Demographics			
Age, years	64.0 (51.0–75.0)	72.0 (56.0–77.0)	0.222
Sex, male	17 (65.4)	14 (38.9)	0.072
Body mass index, kg/m^2	24.4 (22.7–25.7)	24.1 (21.9–26.2)	0.781
Comorbidities and risk factors			
Hypertension	11 (42.3)	21 (58.3)	0.323
Diabetes mellitus	7 (26.9)	11 (30.6)	0.978
Hypercholesterolemia	3 (11.5)	4 (11.1)	1.000
Atrial fibrillation	7 (26.9)	12 (33.3)	0.794
Coronary artery disease	3 (11.5)	3 (8.3)	1.000
Previous stroke	4 (15.4)	5 (13.9)	1.000
Cancer	2 (7.7)	3 (8.3)	1.000
Smoking	10 (38.5)	13 (36.1)	1.000
Alcohol consumption	11 (42.3)	10 (27.8)	0.357
Diagnosis on admission			
Stroke	16 (61.5)	30 (83.3)	0.101
CNS infection	4 (15.4)	4 (11.1)	0.911
Seizure or status epilepticus	4 (15.4)	2 (5.6)	0.392
Demyelinating disease	2 (7.7)	0 (0)	0.335
Clinical status on admission			
Glasgow Coma Scale	12.0 (11.0–14.0)	12.0 (10.0–14.0)	0.868
APACHE-II score	11.5 (9.0–17.0)	15.5 (12.5–19.5)	0.014

MIDA = midazolam; DEX–KET = a combination of dexmedetomidine and ketamine; CNS = central nervous system; APACHE-II = Acute Physiology and Chronic Health Enquiry II; MRI = magnetic resonance imaging

[a]Results presented as numbers (%) or medians (interquartile range).

[Supplementary table 7] Changes of vital signs in the MIDA' and DEX–KET groups

	MIDA' group (n = 26)	DEX–KET group (n = 36)	*P* value
Systolic blood pressure			
Time 0	134.0 (117.0–154.0)	133.5 (124.5–148.0)	0.549
Time 1	129.0 (112.0–147.0)	135.0 (127.0–153.5)	0.140
Time 2	122.0 (108.0–141.0)	127.5 (113.0–140.5)	0.633
Diastolic blood pressure			
Time 0	76.0 (64.0–81.0)	75.0 (61.5–86.0)	0.920
Time 1	76.0 (65.0–81.0)	76.0 (62.0–87.5)	0.612
Time 2	71.5 (65.0–83.0)	70.0 (58.5–80.0)	0.607
Heart rate			
Time 0	86.5 (75.0–96.0)	85.5 (66.0–94.5)	0.284
Time 1	90.5 (78.0–98.0)	79.5 (66.0–92.5)	0.078
Time 2	83.0 (71.0–93.0)	71.0 (59.0–87.5)	0.035
Respiratory rate			
Time 0	18.5 (15.0–20.0)	19.0 (16.5–21.5)	0.367
Time 1	20.0 (18.0–21.0)	19.0 (16.5–23.0)]	0.960
Time 2	19.0 (18.0–20.0)	16.5 (14.0–20.5)	0.051
Oxygen saturation			
Time 0	98.0 (97.0–100.0)	98.0 (96.0–99.5)	0.632
Time 1	97.0 (94.0–98.0)	97.0 (96.0–99.5)	0.207
Time 2	99.0 (98.0–100.0)	98.0 (96.0–99.5)	0.232

[a]Time 0 = immediately before administration of sedative agents; time 1 = immediately after bolus infusion of sedative agents; time 2 = soon after returning to the neurological intensive care unit

[b]Results presented as a median (interquartile range).

[Supplementary table 8] Outcomes and complications according to the MRI sedation protocol in the MIDA' and DEX–KET groups

	MIDA' group (n = 26)	DEX–KET group (n = 36)	*P* value
Outcomes relevant to MRI sedation			
Success in MRI sedation	22 (84.6)	33 (91.7)	0.646
MRI scan time, min	100.0 (87.0–110.0)	53.5 (45.0–60.5)	<0.001
Length of neuro-ICU stay, day	3.0 (2.0–5.0)	4.0 (3.0–6.0)	0.082
Complications following MRI sedation	15 (57.7)	8 (22.2)	0.010
Oxygen desaturation	4 (15.4)	1 (2.8)	0.185
Hypotension	1 (3.8)	0 (0)	0.869
Cardiorespiratory arrest	0 (0)	0 (0)	1.000
Aspiration pneumonia	13 (50.0)	7 (19.4)	0.024

MRI = magnetic resonance imaging; neuro-ICU = neurological intensive care unit.

[a]Results presented as a number (%) or median (interquartile ranges)

[Supplementary table 9] Factors associated with complications following MRI sedation in the MIDA' (n = 26) and DEX–KET (n = 36) groups

	Univariable analysis	*P* value	Multivariable analysis	*P* value
	OR (95% CI)		AOR (95% CI)	
Demographics				
Age, years	1.00 (0.97–1.03)	0.964		
Sex, male	1.15 (0.41–3.22)	0.793		
Body mass index, kg/m^2	0.94 (0.80–1.10)	0.422		
Comorbidities and risk factors				
Hypertension	0.79 (0.28–2.21)	0.647		
Diabetes mellitus	0.62 (0.22–1.74)	0.128		
Hypercholesterolemia	1.31 (0.27–6.47)	0.738		
Atrial fibrillation	0.71 (0.23–2.22)	0.551		
Coronary artery disease	0.31 (0.03–2.83)	0.298		
Previous stroke	0.18 (0.02–1.51)	0.112	0.05 (0.04–0.72)	0.028
Cancer	2.78 (0.43–18.01)	0.285		
Smoking	0.32 (0.10–1.05)	0.060	0.22 (0.05–0.90)	0.035
Alcohol consumption	0.57 (0.18–1.75)	0.323		
Diagnosis on admission				
Stroke	0.24 (0.07−0.78)	0.018		
CNS infection	3.33 (0.72–15.54)	0.125		
Seizure or status epilepticus	1.80 (0.33–9.77)	0.496		
Demyelinating disease				
Clinical status on admission				
Glasgow Coma Scale	0.85 (0.68–1.06)	0.128	0.76 (0.58–1.01)	0.062
APACHE-II score	1.01 (0.92–1.11)	0.866		
MRI sedation, DEX–KET	0.21 (0.07–0.63)	0.006	0.12 (0.03–0.46)	0.002

MRI = magnetic resonance imaging; CNS = central nervous system; APACHE-II = Acute Physiology and Chronic Health Enquiry II; OR = odds ratio; AOR = adjusted odds ratio

[Supplementary table 10] MRI sequence and number of images in the MIDA and DEX–KET groups

	MIDA group (n = 36)	DEX–KET group (n = 36)
DWI	30 (83.3%)	36 (100.0%)
FLAIR	26 (72.2%)	35 (97.2%)
GRE	24 (66.7%)	34 (94.4%)
SWI	11 (30.6%)	10 (27.8%)
MIP	1 (2.8%)	1 (2.8%)
TOF	19 (52.8%)	27 (75.0%)
T1WI	13 (36.1%)	8 (22.2%)
T2WI	15 (41.7%)	8 (22.2%)
T1E	14 (38.9%)	8 (22.2%)
PWI	1 (2.8%)	1 (2.8%)
CE-MRA	8 (22.2%)	6 (16.7%)
Number of MRI images in each patient	150.0 (150.0–357.0)	150.0 (150.0–300.0)

MRI = magnetic resonance imaging; DWI = diffusion-weighted imaging; FLAIR = fluid-attenuated inversion recovery; GRE = gradient-echo imaging; SWI = susceptibility-weighted imaging; MIP = maximum intensity projection imaging; TOF = time-of-flight imaging; T1WI = T1-wegithed imaging; T2WI = T2-weighted imaging; T1E = T1-enhanced imaging; PWI = perfusion-weighted imaging; CE-MRA = contrast-enhanced magnetic resonance imaging

[Supplementary table 11] MRI sequence and number of images in the MIDA' and DEX–KET groups

	MIDA' group (n = 26)	DEX–KET group (n = 36)
DWI	23 (88.5%)	36 (100.0%)
FLAIR	19 (73.1%)	35 (97.2%)
GRE	17 (65.4%)	34 (94.4%)
SWI	9 (34.6%)	10 (27.8%)
MPI	1 (3.8%)	1 (2.8%)
TOF	14 (53.8%)	27 (75.0%)
T1WI	9 (34.6%)	8 (22.2%)
T2WI	11 (42.3%)	8 (22.2%)
T1E	9 (34.6%)	8 (22.2%)
PWI	1 (3.8%)	1 (2.8%)
CE-MRA	4 (15.4%)	6 (16.7%)
Number of MRI images in each patient	150.0 (150.0–355.0)	150.0 (150.0–300.0)

MRI = magnetic resonance imaging; DWI = diffusion-weighted imaging; FLAIR = fluid-attenuated inversion recovery; GRE = gradient-echo imaging; SWI = susceptibility-weighted imaging; MIP = maximum intensity projection imaging; TOF = time-of-flight imaging; T1WI = T1-wegithed imaging; T2WI = T2-weighted imaging; T1E = T1-enhanced imaging; PWI = perfusion-weighted imaging; CE-MRA = contrast-enhanced magnetic resonance imaging

[Supplementary figure 1] New MRI sedation protocol for patients in the neuro-ICU

Ver. August 1, 2016

MRI Sedation Protocol for Patients in the Neuro-ICU

Name: ID: DATE:

1. Inclusion criteria (for MRI sedation)

☐ Agitation (RASS scale ≥1) for recent 24 hr

☐ Physical restraints

☐ Recent failure to neuroimaging study

☐ Recent use of sedative agents for neuroimaging studies

2. Exclusion criteria (for a combination of dexmedetomidine and ketamine)

☐ QTc prolongation >550 ms

☐ Second/third degree AV block

☐ Severe heart failure

☐ Intractable hypertension/hypotension

☐ Allergy to dexmedetomidine or ketamine

3. Sedation regimen

Start following agents 10 minutes before going to the MRI room:
Dexmedetomidine 35 mcg over 10 min + Ketamine 35 mg over 1 min
(Dexmedetomidine in N/S 50 ml & Ketamine in N/S 10 mL)
Fill out this checklist after finishing the infusion and then move the patient to the MRI room.

⇨

If the patient does not cooperate in the MRI room:
Repeated dexmedetomidine plus ketamine, as indicated in the left box

4. Checklist

	Right before administering protocol medication	Immediately after administering protocol medication (= right before moving the patient to the MRI room)	Immediately after returning to the neuro-ICU
RASS scale (-5 ~ +4)			
Systolic BP (mmHg)			
Diastolic BP (mmHg)			
Heart rate (/min)			
Respiratory rate (/min)			
SpO_2 (%)			
☐ Use of additional dose of sedative agents in MRI rooms specify: ( )			

* Notify to the duty doctor of the neuro-ICU if systolic BP < 90 mmHg; systolic BP > 200 mmHg; change of heart rate by more than 40/min; heart rate < 40/min; heart rate > 130/min; or SpO_2 <90%.

5. Success or failure

☐ Protocol success (completion of all MRI sequences as ordered)

☐ Protocol failure (no completion of all MRI sequences as ordered)

[Supplementary figure 2] Flowchart of the patient selection process in the MIDA’ and DEX–KET groups

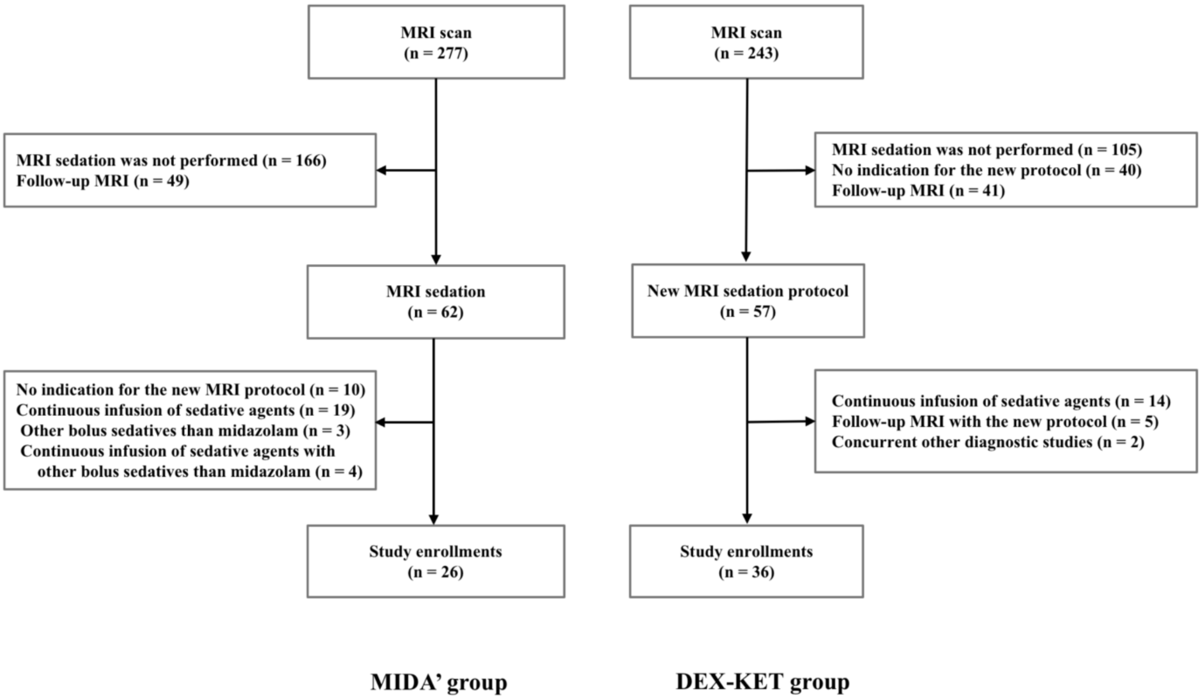

In the left side of the figure, 10 more patients who did not meet inclusion criteria and met exclusion criteria for the new MRI protocol were excluded as compared with Figure 1. Inclusion and exclusion criteria for the new MRI protocol in Supplemental Figure 1.

[Supplementary figure 3] Changes in the level of consciousness before and after administration of sedative agents

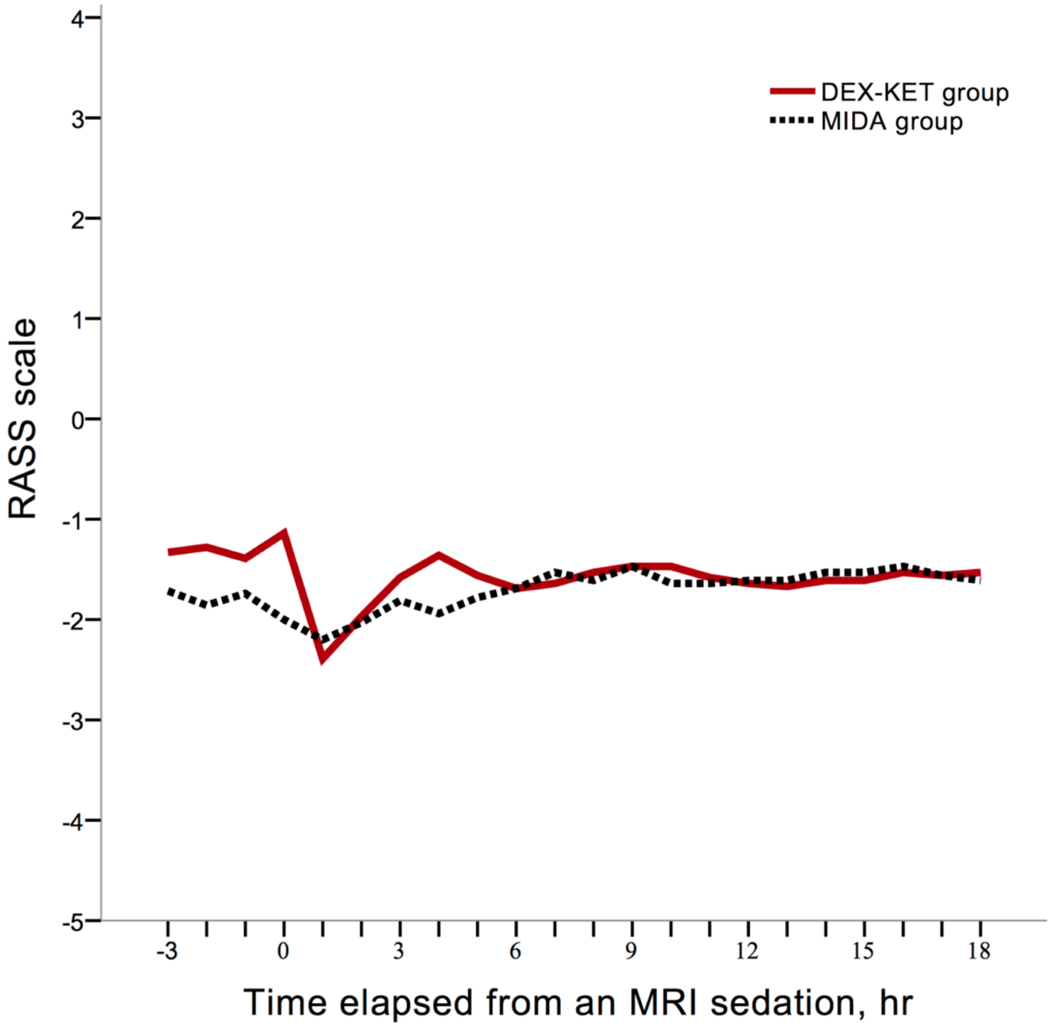

RASS = the Richmond Agitation-Sedation Scale; MRI = magnetic resonance imaging
